# Engineered Extracellular Vesicles for Drug Delivery in Therapy of Stroke

**DOI:** 10.3390/pharmaceutics15092173

**Published:** 2023-08-22

**Authors:** Waqas Ahmed, Muhammed Shibil Kuniyan, Aqil Mohammad Jawed, Lukui Chen

**Affiliations:** 1 Department of Neurosurgery, Neuroscience Center, Integrated Hospital of Traditional Chinese Medicine, Southern Medical University, Guangzhou 510310, China; ahmed.waqas3@gmail.com; 2 School of Medicine, Southeast University, Nanjing 210009, China; shibil1996@gmail.com (M.S.K.); doctorjawedaqil@gmail.com (A.M.J.)

**Keywords:** extracellular vesicles, engineering, stroke, blood-brain-barrier, drug delivery

## Abstract

Extracellular vesicles (EVs) are promising therapeutic modalities for treating neurological conditions. EVs facilitate intercellular communication among brain cells under normal and abnormal physiological conditions. The potential capability of EVs to pass through the blood–brain barrier (BBB) makes them highly promising as nanocarrier contenders for managing stroke. EVs possess several potential advantages compared to existing drug-delivery vehicles. These advantages include their capacity to surpass natural barriers, target specific cells, and stability within the circulatory system. This review explores the trafficking and cellular uptake of EVs and evaluates recent findings in the field of EVs research. Additionally, an overview is provided of the techniques researchers utilize to bioengineer EVs for stroke therapy, new results on EV–BBB interactions, and the limitations and prospects of clinically using EVs for brain therapies. The primary objective of this study is to provide a comprehensive analysis of the advantages and challenges related to engineered EVs drug delivery, specifically focusing on their application in the treatment of stroke.

## 1. Introduction

**Figure 1:**
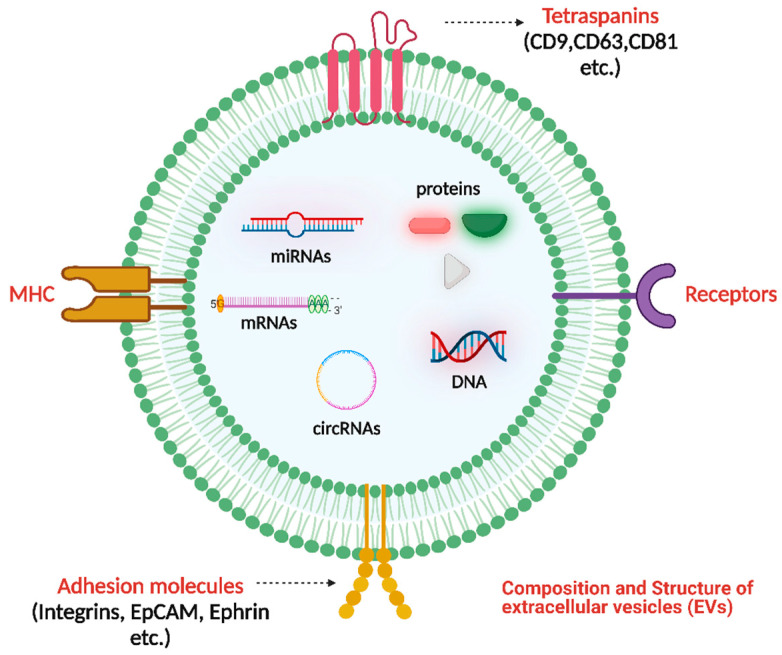
Composition and Structure of EVs. The structure of EVs consists of a phospholipid bilayer that encloses proteins (membrane protein and cargo protein) and nucleic acids. Membrane proteins encompass a variety of molecules, such as tetraspanins (including CD9, CD63, and CD81, among others), adhesion molecules (such as integrins, EpCAM, and Ephrin), the major histocompatibility complex (MHC), and receptors. Nucleic acids encompass DNA and RNA, which consist of various types of RNA molecules, such as messenger RNA (mRNA), microRNA (miRNA), long non-coding RNA (lncRNA), and circular RNA (circRNA). The phospholipid bilayer confers protection to the contents enclosed within. Created with BioRender.com (https://app.biorender.com/illustrations/649b3cd0422a45d3d7cc3c29, accessed date: 7 July 2023).

**Figure 2:**
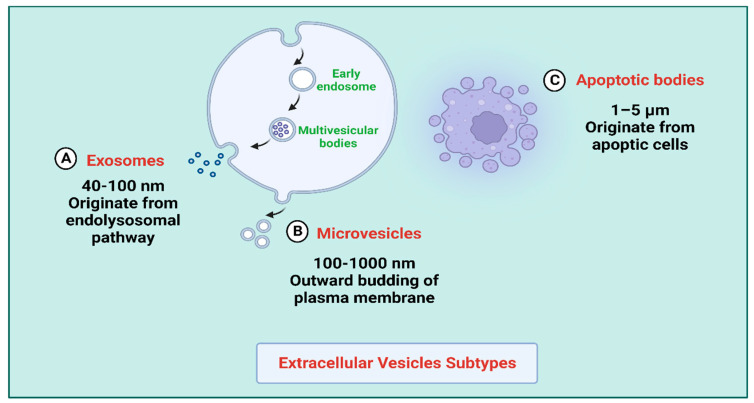
Schematic of EV Subtypes. (**A**) Exosomes and their formation. (**B**) Microvesicles and their formation. (**C**) Apoptotic bodies and their formation. Created with BioRender.com (https://app.biorender.com/illustrations/64d0be423f71706d207a7881, accessed date: 7 August 2023).

EVs are nanoscale heterogeneous structures released constitutively into the extracellular space by nearly all prokaryotic and eukaryotic cell types [1,2,3,4]. EVs are lipidic vesicles that develop in cells naturally. They facilitate cell-to-cell interaction by transferring various bioactive molecules from the mother cell, such as microRNAs, messenger RNAs, long noncoding RNAs (LncRNAs), circular RNAs (circRNAs) proteins, and lipids [5] (Figure 1). They are divided into three distinct categories: (i) Exosomes, (ii) Microvesicles, and (iii) Apoptotic bodies (Figure 2). Exosomes are a minor type of EVs, and prior electron microscopy studies have shown that they have a cup-like structure with a diameter ranging from 40 to 100 nm. During the development of multivesicular endosomes, an inward budding of the endosomal membrane produces exosomes [6]. Microvesicles, the second most significant type of vesicle, are formed when the plasma membrane buds and splits apart, with diameters between 100 and 1000 nm [7]. Apoptotic bodies represent the most abundant type of vesicles, exhibiting a size range of 1–5 μm and a diverse range of morphological characteristics. They are generated during the process of apoptosis. As a result, they possess a wide variety of constituents inherited from their progenitor cells, such as organelles and fragments of DNA [8].

Stroke is a prevalent neurological condition that can lead to permanent disability [9,10]. Annually, approximately 6.7 million individuals globally experience a stroke, with ischemic stroke comprising 87% of all mortalities [11]. The pathophysiological responses that occur after a stroke are complicated, and at present, tissue plasminogen activator remains the most efficacious treatment option for stroke [12,13], effective only when taken within 4–6 h of the onset of symptoms [14,15]. Despite this, due to its limited treatment window, it benefits less than 5% of patients [16]. Recent studies suggest that utilizing anti-inflammatory methods has considerable potential for expanding the time frame for treatment and decreasing severe brain damage following reperfusion [17,18]. Still, there is a constant demand for effective and cutting-edge medications that may prevent ischemia cascades and the diseases they cause.

EVs significantly function in the complex intercellular communication among neurons, glia, and vascular cells. They are essential in regulating homeostasis and influencing the development and prognosis of pathological conditions. EVs are involved in various physiological processes, including the maintenance and restoration of neurons [19], synaptic function [20], neurovascular stability [21], and the preservation of myelination [22]. In recent years, several studies have demonstrated the potential of EVs as nanotherapeutics for treating brain pathologies [23,24,25]. The scientific world has taken a keen interest in this topic, with numerous studies demonstrating the neuroprotective and regenerative effects of natural EVs from various resources [23,26,27].

The present review suggests that engineered EVs can enhance the curative effectiveness of EV-based treatments for stroke. We will explore the curative abilities of natural EVs for neurological applications, including crucial factors for their therapeutic success. We also discuss recent advances in regulating the BBB by EVs and their movement throughout the BBB. In our final part, we summarized the many scientific approaches discovered for modulating the content and surface of EVs, with a particular emphasis on the tactics employed for various therapeutic and targeting drugs.

## 2. Analysis Criteria

A digital database and search tool were utilized to thoroughly review scientific literature, including original articles regarding experimental and observational studies, case series, and reports, among other relevant sources. PubMed, Google Scholar, Scopus, Web of Science, bioRxiv, medRxiv, CNKI, and WanFang Data are central databanks utilized for medical studies. (The latter two databases are particularly significant within the Chinese mainland.) The study analyzed a total of 42 articles that were published or in preprint form between December 2017 and June 2023. A brief overview of the connection between EVs and stroke was found.

## 3. Isolation and Characterization of EVs

The primary challenge in isolating and characterizing EVs lies in their small size and heterogeneous nature. In 2018, the International Society for Extracellular Vesicles updated its “Minimum information for studies of extracellular vesicles” (MISEV) standards, which can be summed up as follows: (I) give a quantitative description of the EV source; (II) show the existence of the functioning bilayers of lipids and describe their purity; (III) use a mixture of EV analysis methods, ideally one optical and one biophysical/biochemical; and (IV) use a solid and accurate experimental design when figuring out how EVs work in the body [28,28].

**Figure 3:**
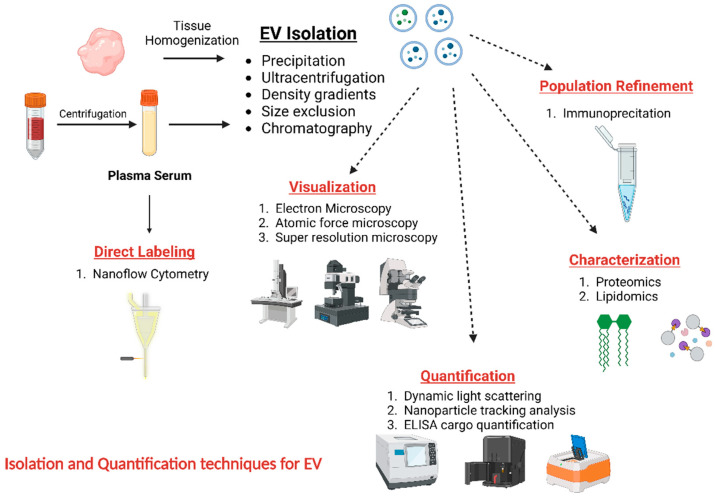
Isolation and quantification techniques for EVs. The diagram illustrates frequently employed methods for analyzing EVs. EVs can be quantified in tissue homogenates and natural fluids, such as urine, saliva, and blood. The isolation of plasma or serum from blood is utilized in this context as an illustrative case. Nanoflow cytometry enables direct labeling and quantifying EVs in various fluid samples. In contrast, EVs have the potential to be separated and subsequently utilized for further analysis. Immunoprecipitation is a technique that can be employed to enhance the specificity of EV populations. EVs can be observed using electron microscopy (EM) or alternative high-resolution microscopy methodologies. Lipidomics and proteomics methodologies can also be utilized to analyze and describe the composition of EVs populations. Ultimately, EV concentration and size measurement can now be achieved through dynamic light scattering and nanoparticle tracking analysis. Additionally, EV products can be measured by employing susceptible protein or RNA assays. Created with BioRender.com (https://app.biorender.com/illustrations/649b163862468f1db106b519, Accessed date: 7 August 2023).

The methodologies used for EV separation exhibit a wide range of diversity. Moreover, every single technique may possess unique parameters and configurations designed to enrich specific subtypes of EVs. The workflow of EV analysis is shown in (Figure 3), along with typical methods for EV size, concentration, and cargo measurement. The selection of the EV separation method, or a combination of methods, is significantly impacted by the research purpose, as well as considerations of time, costs, and applicability. This is particularly important when considering potential clinical applications [29].

## 4. EV Extraction from Biological Specimens

EVs have been extracted from a wide range of biological fluids, such as blood, urine, saliva, breast milk, cerebrospinal fluid, ascitic fluid, gastric juice, bile, sputum, bronchoalveolar lavage, semen, and tears [30,30,31,32,33,34]. They are actively released by cells throughout various tissues and organs, exhibiting their presence in physiological and pathological conditions [35,36]. The primary contributors to the population of blood microvesicles are platelets, which constitute a significant proportion of blood, with EVs ranging from 70% to 90% [35]. In addition to the mentioned hematopoietic cells, many other cell types, including reticulocytes, B lymphocytes, T cells, neutrophils, mast cells, dendritic cells, and macrophages, also play a significant role. Moreover, cells originating from different bodily tissues, such as epithelial cells, also contribute to this process [37]. The use of standardized pre-analytical procedures (Figure 3) is of the greatest significance to minimize the presence of mistakes in the analysis of EVs, mainly when these EVs are obtained from complex body fluids, such as blood. The composition, concentration, and characteristics of EVs produced from biofluids can be influenced by various factors, such as age, gender, ethnicity, body mass index, disease status, medication usage, overall lifestyle, and dietary lifestyles [38]. These variables must be considered and modified across all study participants (patients and any relevant controls).

## 5. Natural EVs for Treating Stroke

The significance of EV signaling within the brain’s framework was initially recorded in the early 1950s through electron microscopy studies [39,40,41,42]. Since then, researchers have discovered that EVs produced by nerve cells, like astrocytes and microglia, play an important role in immune signaling during synaptic plasticity [43,44,45]; in the specialized nature of neural cell interaction [46,47]; and in the diffusion of various neurological diseases, like neurodegenerative diseases and cerebral tumors [48]. Stroke [24], traumatic brain injury (TBI) [49], Alzheimer’s disease (AD) [50], autism [51], and schizophrenia [52] are just a few of the brain disorders where the therapeutic effects of EVs have been described since 2011. Over the past decade, a notable transition has been from cell-based treatments to EVs treatments. Several clinical investigations have exhibited the potential of EVs to protect and regenerate in various therapeutic applications in stroke (Table 1).

### 5.1. EV Origin

**Figure 4:**
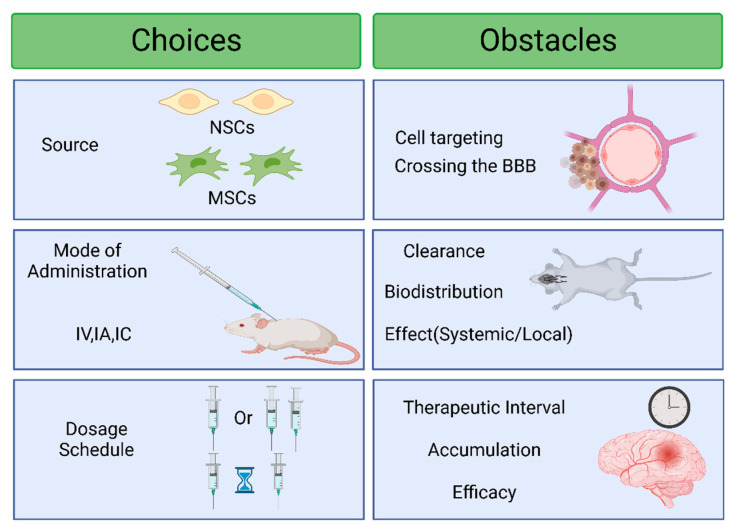
Choices and obstacles while using EVs. The application of EVs originating from diverse cellular origins is commonly observed in managing stroke. The properties of EVs associated with each cell type may have varying levels of tropism for brain vasculature or neuronal cells, which could impact their ability to target the brain effectively. However, a complete evaluation of these properties has yet to be conducted. Crossing the blood–brain barrier (BBB) poses a significant challenge. The method of administration affects the biodistribution and clearance of EVs and can also impact the effect’s nature, i.e., whether it is localized or systematic. Lastly, the dosing schedule can be single or repeated, affecting accumulation and efficacy. Created with BioRender.com (https://app.biorender.com/illustrations/647df1821b2f09af295c29aa, accessed Date: 5 June 2023).

According to current research, the origin of EVs after systemic treatment affects their biodistribution [64]. This aspect has yet to be thoroughly investigated when designing EVs as therapies for stroke. A natural brain stereotype could be employed to enhance the therapeutic benefit of an EV. As far as current knowledge goes, research has yet to be conducted to directly contrast the brain tropism of extracellular vesicles derived from various origins. Research on native EVs for stroke has focused on their therapeutic potential rather than their specific ability to target the brain. According to experimental evidence, the mechanism by which EVs are derived from mesenchymal stem cells (MSCs) targeting damaged areas in the brain may be influenced by inflammatory processes [65]. Previous research has utilized EVs released by neural stem cells (NSCs) sourced from the subventricular area of mice [6,46,66] or from human NSCs [57] that were derived following the division of induced embryonic stem cells (Figure 4). Initial research utilizing mouse NSC-EVs indicated that these EVs are more likely to build in the liver and lung than the brain following intravenous or retro-orbital administration [55].

Substances on their surface mediate the natural targeting capability of EVs [67,68]. Findings from the report of metastatic progression have revealed that the brain tropism of EVs originating from breast tissue cells [68] is determined by expressing Integrin Beta3. Additional research on surface molecules that facilitate brain targeting may offer insights into selecting cell sources or developing engineering techniques to improve cerebral tropism.

### 5.2. Route of Administration

The route of administration is an essential factor to consider when investigating the biodistribution of a drug. This is also applicable to the investigation of EVs. (Figure 4). In a variety of animal experiments, EVs have been given to the animals intracerebrally [69,70,71,72,73], intravenously [74], intranasally [75], intra-arterially, intraperitoneally [76], and via retro-orbital [77] routes. Limited research has been conducted to compare the number of EVs produced in the nervous system through various delivery methods.

Administering EVs through the intra-arterial route is more productive for targeting the brain than the other routes. This is because the EVs are delivered near the brain, which reduces their elimination by other organs in the body [64]. EVs generated from human mesenchymal stem cells isolated from human bone marrow were administered intravenously to mice with acute brain lesions resembling ischemic stroke conditions [78]. Following treatment with EVs, a reduction in the presence of macrophages was observed in the affected area compared to the control group. In addition, there was a decline in the expression of pro-inflammatory cytokines and astrocyte activation.

### 5.3. EV Dose Comparisons

In addition to the delivery pathway and cellular origin, the number of EVs and the schedule of administration are critical factors that significantly impact the therapeutic effectiveness of the treatment (Figure 4). Various doses and administration regimens have been utilized to treat stroke using native EVs (Table 1). In stroke therapy, Aβ oligomers have been reduced in rats and a transgenic mouse model by administering native EVs at concentrations ranging from 10 to 100 μg and 30 to 30 μg, respectively [60,61,63,72,79]. Some studies administer EVs only once, while others administer them multiple times due to the quick elimination of EVs from the infarcted region within twenty-four hours of the initial intravenous injection [80] to achieve sustained brain accumulation. The administration time varied between 2 and 48 h post-ischemic stroke for single-dose therapies and 2 h weekly for multiple-dose regimens [23,60,81]. It has been suggested that reducing systemic inflammation in the blood after a stroke can be achieved by increasing the number of M2-type macrophages and Treg populations and decreasing the number of Th17 cells within 2 h of treatment. This creates a favorable environment for successful brain remodeling [57]. Importantly, from a therapeutic standpoint, higher concentrations of EVs are not always preferable. NPC-EVs or MSC-EVs at an average dose boosted neuronal densities in stroke mice but not at either low or high doses.

### 5.4. Mechanism of Action

**Figure 5:**
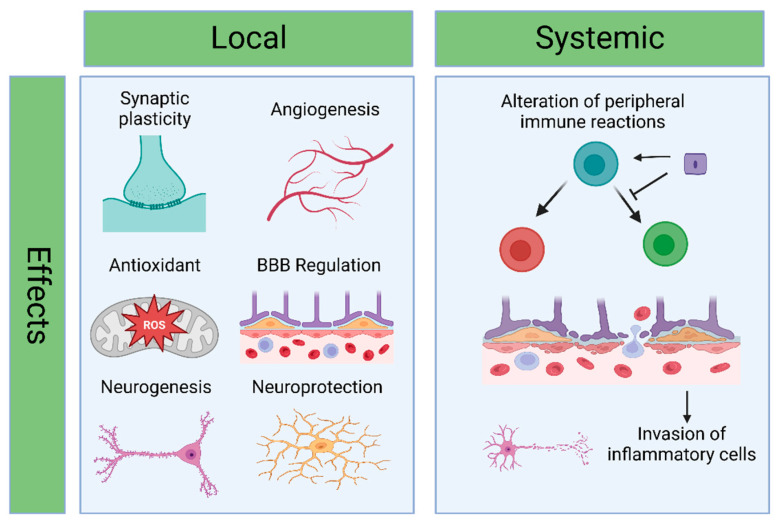
The role of natural EVs in the pathophysiology of stroke. The management of stroke has shown benefits through direct local effects in the brain, such as neuroprotection, neurogenesis, angiogenesis, antioxidant and anti-inflammatory properties, and systemic effects by modulating peripheral immune system responses. These effects may create a favorable environment for cerebral regeneration. Created with BioRender.com (https://app.biorender.com/illustrations/647df0bfe74d4f82a5bc4ade, accessed: 11 June 2023).

The purpose of current study aims to examine the functional benefits of vesicles that are secreted from mesenchymal stem cells (MSCs) [82] and neural stem cells [57], which have been identified in rodents, rats, and pigs [60] models of cerebral ischemia that were caused by the blockage of the middle cerebral artery. It has been demonstrated that MSC-derived EVs have similar outcomes to those described with MSC transplantation in terms of decreasing infarct volume, enhancing functional recovery, increasing angiogenesis and neovascularization [83,84], decreasing astrocyte stimulation [60], and modulating peripheral immune system responses (Figure 5).

Most reported therapeutic effects of EVs in treating stroke are believed to be passive, mediated explicitly through extracranial organs. EVs facilitate a reduction in the overall inflammatory response following stroke, which might decrease leukocyte infiltration in the brain, ultimately reducing blood–brain permeation and neuronal inflammation [57,85]. The study reveals that NSC-EVs can induce the polarization of macrophages toward an M2 genotype with anti-inflammatory properties. Additionally, NSC-EVs were found to increase the population of regulatory T cells while reducing the number of proinflammatory T helper 17 cells [57].

The fact that EVs can cross the BBB and retain their functional cargo has significantly contributed to the development of biomarker research using EVs and their potential application as a vehicle for drug delivery. Alvarez-Erviti et al. showed that mice injected with EVs delivered siRNA to the brain. Dendritic cells were modified to express EVs membrane protein Lamp2b26. Genetically pairing Lamp2b with a CNS-specific rabies virus glycoprotein (RVG) peptide targeted EVs exclusively to the brain [50]. In another study, researchers utilized rats as subjects to investigate the presence of a fluorescently labeled protein specifically expressed in brain tissue. The study found this protein could be detected in microscopic EVs in the rats’ blood. This research study presents empirical support for intercellular communication facilitated by EVs originating from the CNS and disseminating throughout the peripheral tissues [86]. The studies provide evidence that supports the concept of EV crossing the BBB in a bidirectional manner. However, the precise mechanism by which this crossing occurs remains uncertain.

**Figure 6:**
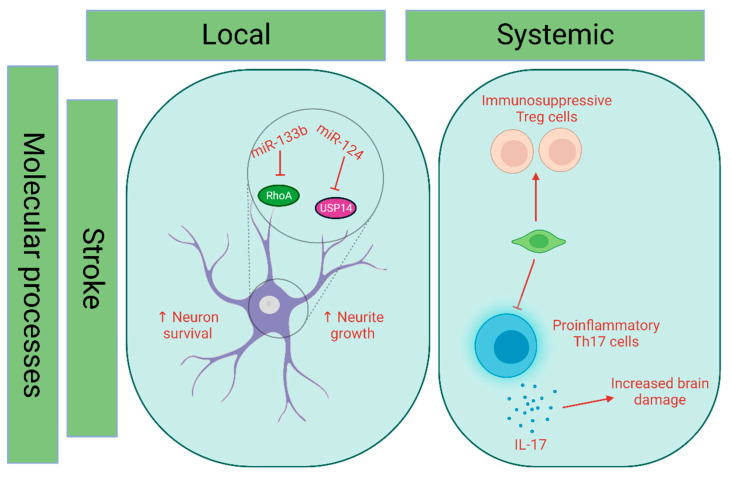
Natural EV modes of action in stroke. The transfer of MiR-133b through EVs originating from mesenchymal stem cells (MSCs) has been found to facilitate the growth of neurites. This effect is achieved through the targeting of the converting protein RhoA. Additionally, miR-124 is linked to increased neuronal viability by targeting USP-14, a ubiquitin-specific protease. Systemically, it has been demonstrated that EVs from NSC reduce pro-inflammatory Th17 cells while improving immunosuppressive Treg cells. Created with BioRender.com (https://app.biorender.com/illustrations/647ca600a95ee7a5e7fd6757, accessed: 11 June 2023).

The therapeutic efficacy of EVs in stroke is primarily attributed to the molecular mechanisms involving microRNAs (miRNAs) present within these vesicles. Typically, in vitro experiments evaluate the direct impact of EVs on neuronal cells. They promote neurite outgrowth in nerve cells, and the inhibition of the growth factor for connective tissue in astrocytes was observed upon exposure to miR-133b-containing EVs released by mesenchymal stem cells (MSCs). This effect was attributed to the suppression of RhoA by the EVs [87,88]. Furthermore, it has been discovered that EVs containing miR-124, released by M2 microglia cells, can increase neuronal survival in vivo. The downregulation of ubiquitin-specific protease 14 is regulated to achieve the desired result. However, whether the effect is direct or systemic is still being determined since there is no apparent connection between transfected cells and the downregulation of ubiquitin-specific protease 14 (Figure 6).

## 6. Neuronal Regeneration via Sustained EV/NP Delivery

Over several decades, extensive research has been conducted on utilizing the body’s inherent regenerative capacity. Numerous studies have investigated the utilization of various therapeutic approaches to induce endogenous regeneration. These approaches include instructive biomaterial scaffolds, EVs, nanoparticles (NPs), small molecules, and other similar interventions [89]. Therapeutic EVs have been utilized for potential regenerative uses primarily due to their adaptability in modifying size and capacity to serve as carriers for transporting drugs, growth factors, tiny molecules, or genetic information [90,91].

Nerve injuries pose significant clinical challenges as a result of their inherent limitations in terms of regenerative capacity. Conventional treatments for nerve injuries have traditionally relied on autologous nerve grafts. However, these grafts are gradually being supplanted by artificial nerve guidance conduits (NGCs) due to limitations, such as the limited availability of nerve grafts, the need for multiple surgeries to isolate donor grafts, and the potential risk of developing neuromas. The primary objective of a recent research study on managing peripheral nerve injury was to explore the potential of targeted therapeutics. This was achieved by utilizing alginate hydrogels composed of laminin-coated poly(l-lactide-co-glycolide) (PLGA) conduits. These conduits were designed to contain a mixture of gold nanoparticles (NPs), brain-derived neurotropic growth factor (BDNF), and adipose-derived stem cells [92].

**Figure 7:**
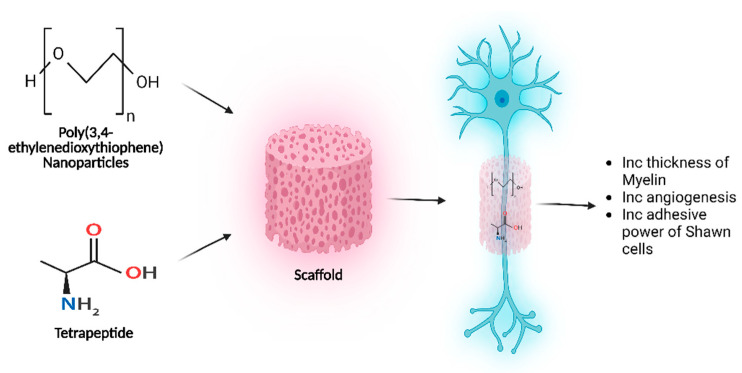
EV/NP. Transport Neural Remodeling. Poly(3,4-ethylenedioxythiophene) modified with tetrapeptide was administered utilizing a biocompatible chitin scaffold. In an in vivo model, myelin thickness increased, indicating nerve regeneration. At the injury’s site, Schwann cell adhesiveness and angiogenesis increased. Created with BioRender.com (https://app.biorender.com/illustrations/64a80816d2fc5a3659e189a9, accessed: 7 July 2023).

A separate investigation examined the administration of conductive poly(3,4-ethylenedioxythiophene) nanoparticles/EVs modified with cell adhesive tetrapeptide. These nanoparticles were delivered using a biocompatible chitin scaffold. The study’s results indicate that the conductive scaffold exhibited high porosity and compatibility with biological systems. Upon introduction into an in vivo model, a notable enhancement in nerve regeneration was observed, as evidenced by an increase in the regenerated myelin’s thickness and the muscle fibers’ area. An augmentation in the adhesive capacity of Schwann cells, along with enhanced angiogenesis, was also noted at the site of the injury (Figure 7). Hence, the utilization of this chitin scaffold with electrical activity has been proposed as a promising alternative for a nerve guidance conduit, as well as a suitable material for the administration of therapeutics to facilitate nerve regeneration [93].

## 7. Drug Loading Techniques

**Figure 8:**
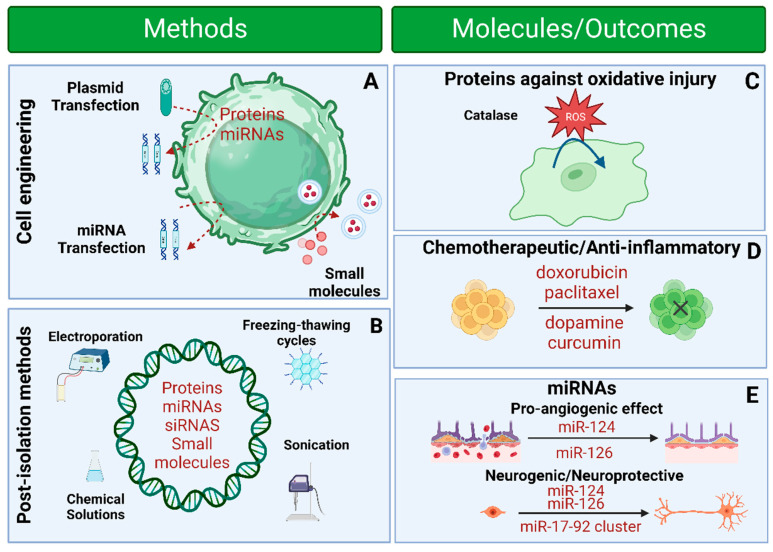
Engineering strategies for modifying EV content. Cellular engineering techniques can regulate EVs, including altering primitive cells or directly loading them via various post-isolation methods. (**A**) Cell engineering uses genetic manipulation techniques, such as plasmid transfection or enriching cells with miRNAs or small compounds, to load a parent cell indirectly. (**B**) Electroporation, sonication, freeze–thaw cycles, and chemical agents modulate isolated EVs post-isolation. Therapeutic substances and loading efficiency determine the optimum EV modulation strategy. EVs cargo manipulation can treat stroke by acting on cargo type. (**C**) Proteins. (**D**) Small molecules. (**E**) miRNAs. Created with BioRender.com (https://app.biorender.com/illustrations/647dfe244f0c62bb59ac8cb8, accessed: 6 June 2023).

The effectiveness of EVs as a new class of nanocarriers arises from their unique qualities as information carriers, including their inherent homing ability, widespread distribution in biological fluids, biological compatibility, cell-specific targeting, non-immunogenicity, and easy penetration through physiological barriers. Pre-loading and post-loading are the two primary strategies for transferring cargo into EVs. Pre-loading refers to loading cargo into parent cells before the isolation of EVs, which releases o EVs that are loaded with cargo. Post-loading refers to loading cargo directly into EVs using either passive or active means after EVs have been isolated (Figure 8). Table 2 provides a comprehensive overview of various cargo loading tactics and procedures employed for extracellular vesicles, along with their advantages and disadvantages.

## 8. Engineered EVs for Stroke Treatment

Despite advancements in the preclinical application of endogenous EVs as therapeutic agents for stroke over the past decade, additional enhancements are required to optimize their therapeutic efficacy and expedite their clinical implementation. The first involves the biological effects of EVs. The EVs of native origin exhibit heterogeneity, even if obtained from an identical cell resource. Therefore, concentrating the EV material into a singular therapeutic entity with the most potent neuronal activity can enhance the efficacy of their treatment. The second is related to the focus on EV targeting. After being administered systemically, very few EVs (usually less than 5%) end up in the brain [109,110,111]. To have the most significant local or direct effect on the brain, we need to make strides in the surface engineering of EVs to improve their range and target particular cell membrane receptors. On the other hand, assessing the in vivo targeting and therapeutic mechanisms of EVs demands the creation of analytical and imaging platforms that are highly sensitive and have high resolution, respectively.

**Figure 9:**
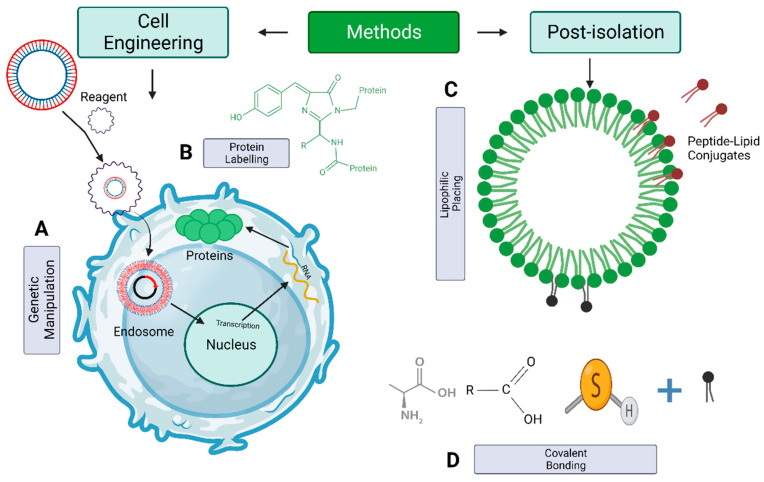
Surface modulation of EVs. The modulation of EV surfaces can be accomplished by genetically modifying the cells that produce them. (**A**) Protein plasmids or (**B**) Protein-residues (**C**) directly conjugated to lipids that are then integrated into EV membranes. (**D**) Bio-orthogonal chemistry identifies extracellular vesicle functional groups. Created with BioRender.com (https://app.biorender.com/illustrations/6480cf6ae6947f82704ea72e, accessed: 11 June 2023).

Consequently, engineering techniques to modify EV bioactivity [27,112,113,114,115,116,117,118,119,120,121], targeting [122,123,124,125,126,127,128,129,130], and tracking [123] have been created to tackle past difficulties. The earlier approaches can be implemented in EVs after isolation or within the cells responsible for EV production. This can be achieved through the use of gene editing [112], metabolic and residue-specific protein packaging [131], or the process of incubating cells with external compounds [132] or nanoparticles [133] (Figure 8 and Figure 9; Table 2 and Table 3). Numerous techniques are available for rapidly and efficiently engineering EVs with functional groups, genetic material, biologically active proteins, and peptides. However, the development of methods that do not adversely affect the function of EVs remains a challenging task. The present section investigates various methodologies, including genetics, exogenous delivery, and chemically inspired techniques, that can alter EVs’ outside appearance and composition to facilitate drug delivery to the brain.

### 8.1. Modulation of Content

Over the past five years, a remarkable increase in the number of described techniques for loading functional molecules into EVs has been seen [27,116,118,142,143]. The predominant approach employed in such methods involves genetic modification via plasmid transfection of the cells that secrete extracellular vesicles, thereby effectively regulating the composition of the EVs [27,114,116,119,144]. Plasmid transfection can be accomplished through electroporation or incubation with transfection reagents [117]. Therefore, EVs have modulated the expression of genes in human disease models, both in vitro and in vivo, by incorporating functional proteins, mRNAs, microRNAs (miRNAs), and other short noncoding RNAs [119,145]. The direct modulation technique has been implemented in manipulating isolated EVs to incorporate small drug molecules and for encapsulating proteins and small amounts of non-coding RNA within EVs (Figure 8) [106,146,147,148]. When implementing these strategies, it is essential to consider certain factors. The molecules’ size determines the material type that EVs can transport. Numerous molecules, such as miRNAs or small drugs, can be encapsulated within EVs. However, the capacity of EVs is restricted when it comes to larger molecules such as mRNA or proteins. Furthermore, a more significant amount of cargo sometimes equates with increased biological value. Additionally, genetically modified EVs’ effectiveness on the target cell is influenced by internalization efficiency, intracellular trafficking, and channels altered by the EV-based molecules.

The fact that these studies were performed on animal models is the only thing that could be considered a limitation. On the other hand, there is no evidence of this therapeutic possibility in the human field due to the challenges involved in the preparation of engineered EVs and the challenges related to the methods of administration needed to achieve a significant enough result to be considered a valid therapeutic strategy. Although there are plenty of issues to be resolved, the development of standardized techniques and guidelines for EV engineering, isolation, and storage has brought EV-based therapies closer to being utilized for stroke and other neurological illnesses (Table 4).

#### 8.1.1. Nanoparticle

EVs are being utilized to enclose tiny therapeutic particles, typically comprising a medicinal substance, with sizes ranging from 10 nm [123] to 150 nm [152]. This approach aims to enhance the transportation of such nanoparticles across the blood–brain barrier (BBB). The loading of EVs can be achieved through two distinct processes: The first involves isolating the EVs and subsequently subjecting them to an electroporation procedure with nanoparticles [123]. The second process entails transfecting EV-secreting cells with nanoparticles and loading the EVs [68]. Recent research has indicated that EVs exhibit comparable therapeutic efficacy to stem cells derived from ischemic stroke treatment [56,81,153,154]. One central area for improvement in utilizing exosomes is the inability to target the ischemic lesion within the brain specifically. It has been found that using magnetic nanovesicles (MNVs) made from mesenchymal stem cells (MSCs) and filled with iron oxide nanoparticles makes it easier to target the area of the brain that is ischemic. This is achieved by applying an external magnetic field, which facilitates magnetic navigation. Using magnetic navigation resulted in a 5.1-fold increase in the EVs’ capacity to target the ischemic lesion specifically. The MNVs were found to have a significant impact on reducing the infarct volume and enhancing motor function. Additionally, they were observed to stimulate a defensive response, promote vascular development, and prevent cell death in cerebral ischemia [88,155,156,157].

An animal stroke model utilized gold nanoparticles coated with glucose to facilitate non-invasive neurological imaging and EV tracking. This approach aided in identifying the most effective administration route and size parameter [158,159]. The diagnosis and treatment of numerous neurological disorders are impeded due to the limited ability of diagnostic agents to penetrate the blood–brain barrier (BBB) [160]. Following the idea of the Nature Biotechnology Group, which involved the production of EVs as vehicles for genetic treatment with selective targets in the brain [50], gold-coated vesicles targeted brain cells by enhancing nanoparticle permeability to cross the BBB [161].

#### 8.1.2. Proteins

Determining the appropriate approach for encapsulating therapeutic proteins within EVs is based on their intended utilization. The method of transfecting cells that secrete extracellular vesicles (EVs) with plasmids is utilized to produce enzyme-loaded EVs, including catalase [106], Cre-recombinase [117], and the lysosomal enzyme tripeptidylpeptidase-1 (TPP-1) [76]. Following a stroke, there is a change in the proportions of pro-inflammatory and anti-inflammatory proteins, impacting the size of the infarct and the individual’s functional outcome [162,163]. EVs containing IFN-Ɣ have been utilized in treating neurodegenerative conditions such as multiple sclerosis. The study demonstrated that IFN-Ɣ-stimulated extracellular vesicles (EVs) decreased demyelination and neurological inflammation in a murine model [158,164]. They investigated the impact of TNFα and interleukin-1β cytokines on the molecular composition and release of astrocyte-derived extracellular vesicles. The findings indicate that vesicles derived from astrocytes treated with TNFα and IL-1β contained a high concentration of miR-125a-5p and miR-16-5p, which are known to target proteins that regulate neurotrophin signaling.

Furthermore, it was noted that EVs injected with cytokines reduced the neuronal expression of NTKR3 and Bcl2 [165]. The inflammatory cytokine IL-1β, which affects the brain’s inflammatory response to injury, was administered into the brain. An IL-1β injection into the striatum led to more Ly6b+ leukocytes going to the lesion site and more circulating vesicles in the plasma of mice compared to controls. IL-1β also caused astrocytes to release EVs, which quickly passed the BBB [166].

An additional study examined the pro-angiogenic effects of microglial BV2 cells that were polarized by IL-4 and lipopolysaccharide. The cells polarized by IL-4 demonstrated an increase in endothelial cell tube formation through the secretion of EVs. Furthermore, the miRNA-26a profile was observed to be higher in comparison to the LPS-polarized group [167]. Researchers found that preconditioning neural stem cells with IL-6, a proinflammatory cytokine that promotes prosurvival signaling, reduced ischemic injury in a mouse stroke model [167,168].

#### 8.1.3. Small Non-Coding RNAs

The study of EVs in the context of nervous system disorders is a relatively developing area of research. The blood–brain barrier (BBB) is formed by the tightly joined endothelial cells of brain capillaries, which effectively limit the passage of small molecules that are lipid-insoluble (90–98%) into the brain [132,169,170]. Two distinct approaches can be employed for the loading of non-coding RNAs in EVs: (I) chemical (e.g., chemically induced transfection) [106,171] or physical (e.g., electroporation) [50,132] strategies following EV separation; and (II) the introduction of plasmid-encoded non-coding RNAs or non-coding RNAs directly into EV-secreting cells through transfection [113,119,149].

The researcher Yang et al. employed EVs to transport circular RNA to the ischemic area of stroke. The study’s authors used a targeted approach to deliver Circ-SCMHI RNA to neuronal cells. This was achieved by expressing RVG peptides on exosome membranes, which served as a cargo-delivering vehicle system for the Circ-SCMHI RNA of interest. The RVG-circSCMH1-EVs have been found to enhance neuronal plasticity through their binding to MeCP2 and subsequent upregulation of downstream gene expression (Mobp, Igfbp3, Fxyd1, and Prodh). This mechanism is believed to contribute to the maintenance of proper brain function. The administration of RVG-CircSCMH1-EVs via intravenous injection enhanced motor recovery, digit movement, and functional recovery in rodent and non-human primate experimental models. The authors proposed that RVG-CircSCMH1-EVs may possess a broader therapeutic window compared to existing treatments, as it could be administered up to 24 h following the onset of stroke [172].

MiR-124 is recognized for its propensity to promote neuronal development in the developing and mature brain. EVs containing miR-124 facilitate the acquisition of neuronal identity by cortical neural progenitors and confer post-ischemic recovery by promoting reliable cortical neurogenesis [132]. The BACE1 gene was effectively knocked down through siRNA delivery via EVs. This resulted in a significant decrease in β-amyloid levels in the brains of mice with wild-type mutations [50]. MiRNA-210 exhibits promising prospects for enhancing angiogenesis in the context of brain area restoration after ischemic events. The enhancement of miRNA-210 expression resulted in a more excellent restoration of function after stroke. The delivery of miRNA-210 to the ischemic area was achieved by linking EVs with the c(RGDyK) peptide and adding miRNA-210.

This resulted in an upregulation of miR-210 at the ischemic site and increased the expression of integrinβ3, vascular endothelial growth factor, and CD34, significantly improving animal survival rates [130].

#### 8.1.4. Neurotrophic Factors (NTFs)

The neurotrophic factor is being researched as a protective factor in neurological diseases [173,174]. They control the development of neural stem cells, which are accountable for restoring neurological function and repairing vascular damage caused by stroke [175,176]. Neurotrophic factors (NTFs) are a promising therapeutic option for stroke repair. Their diverse neuroprotective functions following ischemic events have been acknowledged [177,178]. The clinical application of NTF is currently not feasible due to the absence of an effective method for delivering it systemically to the ischemic area. Brain-derived neurotrophic factor (BDNF) is extensively studied for its neuroprotective and anti-inflammatory effects. BDNF was packed within naive exosomes and administered intravenously to rats to combat brain inflammation [179]. MiR-206 knockdown EVs reduced early damage to the brain by increasing BDNF levels following the treatment [180]. This suggests that bioengineering vesicles with BDNF could help with brain diseases like stroke. More research is needed to understand how EVs and NTFs work in stroke treatment. It would be highly beneficial to prioritize exploring this area in the future.

## 9. Conclusions and Future Prospective

The domain of EVs is presently considered a rapidly advancing field in fundamental science and applied research [181]. EVs derived from various cellular origins have demonstrated therapeutic efficacy in stroke. Over the past decade, significant progress has been achieved in the field (i) by demonstrating the curative value of specific groups of EVs in preclinical studies for cognitive and behavioral brain diseases; (ii) by discovering the systemic [57] and local [61] actions that characterize EV brain regeneration processes; (iii) by showing the effects of EVs in neurogenesis [132], neural protection, angiogenesis, and cerebral remodeling; and (iv) by describing how EVs are transported through the BBB [132,182]. Based on empirical evidence, EVs are swiftly eliminated from the brain, leading to a significant absence of EVs within a short time. So, experts are working on bioengineering EVs to improve their half-life in circulation, make them more available at the disease site, make it easier to deliver them to specific cells, and use them to provide therapeutic molecules or regenerative medicine.

According to a study [57], treating ischemic stroke with innate EVs resulted in particular outcomes, primarily attributed to the alteration of the immune response rather than a localized effect in the brain. However, high growth in the lesion area could produce a greater neuroprotective and pro-angiogenic impact. Numerous investigations have been conducted, primarily in vitro, wherein the culture environment may influence the EV’s biochemical and biophysical characteristics. Consequently, further research is needed to comprehend the engineered EVs physiological impacts on human well-being comprehensively.

In short, EVs possess significant potential for therapeutic applications. Clinical trials have exclusively involved endogenous EVs, while engineered EVs designed to target the brain have yet to undergo clinical testing. This is primarily due to challenges associated with the large-scale production of EVs and the production of EVs with similar characteristics. Collectively, EV-derived therapeutics and diagnostics have exhibited encouraging outcomes in diverse phases of clinical trials. Nevertheless, most clinical trials on EVs are underway, and the available published data regarding their current progress or results could be much better.

Translating modified EVs into clinical settings presents significant obstacles, including restricted techniques for versatile isolation and purification, EV variability, poor storage conditions, potential immunogenic adverse effects, and batch-to-batch variations. Therefore, much work must be done to utilize EVs’ therapeutic effects properly.

## Figures and Tables

**Table 1:** Natural EVs for the treatment of stroke.

Disease	Model	EV Source	Route	Dose	Outcome	Reference
Stroke	MCAO in mouse	Human MSCs	IV	Multiple administrations	↑ Neurogenesis ↑ Angiogenesis	[53]
		M2 Microglia	IV	100 μg	↑ Neuron protection ↓ Volume of infarction	[54]
		Mouse NSCs	IV	100 μg	↑ Availability of astrocytes ↓ Volume of infarction	[27]
		Mouse NSCs and MSCs	RO	1–100 μg (multiple administrations)	↓ Impaired motor coordination ↑ Neuro-regeneration	[55,56]
	TE-MCAO in mouse	Human NSCs	IV	2.7 × 10^11^ EVs/kg (multiple administrations)	↓ Cerebral atrophy ↑ Motor recovery	[57,58]
	MCAO in rat	Porcine MSCs	IV	100 μg	↑ Functional recovery ↓ Volume of infarct ↑ Angiogenesis	[59]
		Human MSCs	IA	200 μg/kg	↑ Functional recovery ↓ Volume of infarct ↑ Angiogenesis	[60]
		Rat NSCs	ICV	30 μg	↑ Neural protection ↓ Microgliosis ↓ Size of infarct ↓ Behavioral deficits	[61,62]
		Rat MSCs	ICV	100 μg	↓ Size of infarct ↑ Functional recovery	[63]
		Rat MSCs	IV	100 μg	↑ Neuron transformation	[24]

**MSCs**—Mesenchymal stem cells; **IV**—intravenous injection; **MCAO**—middle cerebral artery occlusion; **NSCs**—neural stem cells; **TE-MCAO**—thromboembolic middle cerebral artery occlusion; **RO**—retro-orbital; **ICV**—intracerebroventricularly. ↑ Increase, ↓ Decrease.

**Table 2:** Methods for loading EVs with drugs.

Loading Strategies	Loading Methods	Advantages	Disadvantages	Reference
Pre-loading	Co-incubation	1. Simple 2. Cost-effective 3. EV-friendly	1. Low encapsulation efficiency 2. Strict cargo selection	[94,95,96]
	Transfection	Target molecule overexpression	1. Time-consuming 2. Highly dependent on cell viability 3. Potential toxicity and genetic changes	[97,98]
Post-loading	Co-incubation	1. Easy operation 2. No extra equipment is required 3. Minimal destruction to EVs	1. Low loading efficiency 2. Limited variety	[99,100,101]
	Electroporation	1. Effective loading efficiency 2. Loading of large biomolecules	1. Affect EVs integrity 2. Risk of EVs aggregation 3. Heat can cause damage	[102,103]
	Sonication	High loading efficiency	1. EVs membrane degradation 2. EV aggregation risk	[104,105]
	Freeze–thawing cycle	1. Cost-effective 2. Applicable for most cargoes	1. Low loading efficiency 2. EVs membrane damage 3. EVs aggregation risk	[106,107]
	Surfactant administration	1. Affordable 2. Applicable for most cargoes	EVs surface potential and functionality may be altered	[76,108]

**Table 3:** Techniques for modifying EVs cargo in stroke.

Origin of EVs	Method	Model	Result	Reference
**Rat MSCs**	miR-17-92 cluster overexpression	MCAO rat model	↑ Neurogenesis ↑ Neurological function	[119]
**Rat MSCs**	miR-133b overexpression	MCAO rat model	↑ Neuroprotection	[116]
**Human ADSCs**	miR-126 overexpression	Rat MCAO	↑ Neurogenesis ↑ Angiogenesis ↓ Inflammation	[113,134]
**Mouse EPCs**	miR-126 overexpression	Mouse MCAO	↓ Infarct size ↑ Neurogenesis ↑ Angiogenesis	[135]
**Mouse MSCs**	Diffusion of curcumin-loaded EVs	Mouse MCAO model	↓ Inflammation ↓ Neuronal apoptosis	[122]
**Human MSCs**	Diffusion of leucocyte-loaded EVs	Mouse MCAO model	↓ Brain leukocyte infiltration ↑ Neuroprotection	[136]
**Rat-blood-derived EVs**	PCSK9 overexpression	Mouse ICH model	↑ Neuroprotection ↑ Myelination ↑ Angiogenesis	[137]
**Human umbilical cord blood (UCB)–MSC-derived EVs**	Diffusion of BDNF-Loaded EVs	Rat IVH model	↑ Neuroprotection ↓ Inflammatory response/ Apoptosis/ ↑ Myelination and neurogenesis	[138]
**Bone-marrow–MSCs derived EVs**	miR-21-5p overexpression	Rat SAH model	↓ Neuronal apoptosis ↑ Neuroprotection	[139]
**BMSC-derived EVs**	miR-183-5p overexpression	Rat model of db-ICH	↓ Neuroinflammation ↓ Neurological deficit	[140]
**EVs from angiotensin-converting enzyme 2 (ACE 2)**	Endothelial progenitor cells overexpression	Mouse ICH model	↓ Decreased hemorrhage volume ↓ Brain edema Improved Neurological Deficit Score (NDS)	[141]

**MSCs**—Mesenchymal stem cells; **ADSCs**—adipose-derived stem cells; **EPCs**—endothelial progenitor cells; **EVs**—extracellular vesicles; **MCAO**—middle cerebral artery occlusion; **ICH**—intracerebral hemorrhage; **IVH**—intraventricular hemorrhage; **SAH**—subarachnoid hemorrhage; **BMSCs**—bone marrow mesenchymal stem cells; **db-ICH**—diabetic intracerebral hemorrhage. ↑ Increase, ↓ Decrease.

**Table 4:** EV cargo modification in other neurological conditions.

Origin of EVs	Method	Model	Result	Reference
**Mouse embryonic fibroblasts**	Cre recombinase enzyme overexpression	Transgenic mouse model	Intranasal transport of brain-active proteins	[117]
**Human HEK-293 T**	Overexpression of the catalase enzyme	PD mouse model	↓ Neuronal inflammation	[112]
**Mouse astrocytes**	Transfection with lincRNA-Cox2-siRNA	In vitro/in vivo lincRNA-Cox2 knockout model Intranasal	↓ Expression of lincRNA-Cox2; LPS-induced microglial proliferation	[114]
**Human astrocytes**	Transfection with lincRNA-Cox2-siRNA	In vitro/in vivo lincRNA-Cox2 knockout model Intranasal	Microglial phagocytic activity restored	[149]
**Mouse macrophages**	Transfection with curcumin	Rat AD model	↑ Neuron survival; ↓ Tau phosphorylation	[118]
**Mouse macrophages**	EV loading with catalase: sonication, extrusion, or saponin	Mouse PD model	↓ Oxidative stress ↑ Neuron survival	[106]
**Human ESCs**	EV loading with paclitaxel: diffusion	Orthotopic mouse xenografts	↑ Accumulation at the glioma spot ↑ Mouse survival	[147]
**Mouse BECs**	EV loading with paclitaxel or doxorubicin: diffusion	Xenotransplanted brain cancer zebrafish model	↑ Brain cancer cell elimination	[148]
**Mouse blood serum**	EV loading with dopamine: diffusion	Mouse PD model	↑ Dopaminergic neurogenesis ↑ Symptomatic performance	[150,151]

**PD**—Parkinson’s disease; **AD**—Alzheimer’s disease; **ESCs**—embryonic stem cells; **BECs**—brain endothelial cells. ↑ Increase, ↓ Decrease.
